# Angiotensin II Requires Zinc and Downregulation of the Zinc Transporters ZnT3 and ZnT10 to Induce Senescence of Vascular Smooth Muscle Cells

**DOI:** 10.1371/journal.pone.0033211

**Published:** 2012-03-12

**Authors:** Nikolay Patrushev, Bonnie Seidel-Rogol, Gloria Salazar

**Affiliations:** Division of Cardiology, Department of Medicine, Emory University School of Medicine, Atlanta, Georgia, United States of America; Northwestern University, United States of America

## Abstract

Senescence, a hallmark of mammalian aging, is associated with the onset and progression of cardiovascular disease. Angiotensin II (Ang II) signaling and zinc homeostasis dysfunction are increased with age and are linked to cardiovascular disease, but the relationship among these processes has not been investigated. We used a model of cellular senescence induced by Ang II in vascular smooth muscle cells (VSMCs) to explore the role of zinc in vascular dysfunction. We found that Ang II-induced senescence is a zinc-dependent pathway mediated by the downregulation of the zinc transporters ZnT3 and ZnT10, which work to reduce cytosolic zinc. Zinc mimics Ang II by increasing reactive oxygen species (ROS), activating NADPH oxidase activity and Akt, and by downregulating ZnT3 and ZnT10 and inducing senescence. Zinc increases Ang II-induced senescence, while the zinc chelator TPEN, as well as overexpression of ZnT3 or ZnT10, decreases ROS and prevents senescence. Using HEK293 cells, we found that ZnT10 localizes in recycling endosomes and transports zinc into vesicles to prevent zinc toxicity. Zinc and ZnT3/ZnT10 downregulation induces senescence by decreasing the expression of catalase. Consistently, ZnT3 and ZnT10 downregulation by siRNA increases ROS while downregulation of catalase by siRNA induces senescence. Zinc, siZnT3 and siZnT10 downregulate catalase by a post-transcriptional mechanism mediated by decreased phosphorylation of ERK1/2. These data demonstrate that zinc homeostasis dysfunction by decreased expression of ZnT3 or ZnT10 promotes senescence and that Ang II-induced senescence is a zinc and ROS-dependent process. Our studies suggest that zinc might also affect other ROS-dependent processes induced by Ang II, such as hypertrophy and migration of smooth muscle cells.

## Introduction

Aging is associated with physiological changes that increase predisposition to cardiovascular diseases [1]. For example, increases in inflammatory responses with age promote atherosclerosis [2], which is thought to result from age-related dysfunction of the vascular endothelium and smooth muscle cells [3]. Cellular senescence, a hallmark of mammalian aging, is a process of permanent cell cycle arrest involving changes in gene expression and cell morphology [4], such as increase in the expression of senescence-associated β-galactosidase (SA-β-gal) and increase in cell size [5]. Senescent vascular cells in culture present similar changes to the ones observed in aged arteries, such as an increase in ROS levels in vascular smooth muscle cells (VSMCs) [6]. Senescent VSMCs positive for SA-β-gal have been found in arteries of old animals [7] and in atherosclerotic plaques [8], indicating that cellular senescence could contribute to *in vivo* vascular aging [9] and atherosclerosis [10]. Thus, the study of molecular mechanisms regulating cellular senescence *in vitro* is important to our understanding of age related pathologies like atherosclerosis.

Angiotensin II (Ang II) is a potent mediator of vascular disease including atherosclerosis and the metabolic syndrome [11]. Ang II signaling pathways become activated with age and contribute to the development of atherosclerosis [12] as well as vascular senescence *in vivo* [13], [14] and *in vitro* in VSMCs [14]. Moreover, disruption of the Ang II type 1 receptor promotes longevity [15], suggesting that prevention of Ang II signaling is not only beneficial to prevent cardiovascular disease but also to delay the aging process. Ang II induced-senescence involves a p53/p21-dependent pathway in VSMCs [14]. However, the molecular mechanism is not fully understood and whether other processes that are also disrupted by age could modulate this pathway has not been investigated.

Among the changes induced by age, zinc deficiency is common in the elderly [16]. Zinc is an important nutritional factor contributing to the function of the immune system, metabolic function and antioxidant capacities. Zinc supplementation protects from oxidative stress in cells in culture [17] and in animal models [18]. On the other hand, zinc deprivation increases oxidative stress, induces apoptotic cell death [19] and influences renal and cardiovascular disease [20]. Importantly, zinc deficiency has been postulated as a risk factor in the development of atherosclerosis [21] yet the cellular and molecular basis of this association remains largely unexplored. Since vascular senescence is increased by age and Ang II, and zinc homeostasis dysfunction is also increased by age, we hypothesize that zinc homeostasis regulatory mechanisms and Ang II signaling pathways converge to promote vascular senescence.

Zinc homeostasis is tightly regulated by the expression of metallothioneins (MTs) and membrane proteins that buffer and transport zinc, respectively. Zinc transport and distribution are carried out by two families of zinc transporters [22]. Fourteen members of the Zips (Zrt/Irt-like proteins)/SLC39 (solute carrier 39) family of zinc importers work to increase cytosolic zinc by importing zinc from extracellular or from intracellular compartments. In contrast, ten members of the ZnT (zinc transporter)/SLC30A family of zinc exporters work in the opposite direction to reduce cytosolic zinc concentration by moving zinc out of the cell or into various intracellular compartments. Expression of members of the ZnT family is decreased by age [23] suggesting that ZnT family members could be relevant in age-related dysfunction of zinc homeostasis and cardiovascular diseases, but virtually nothing is known about the expression profile of ZnTs or about the regulation of zinc homeostasis in the vasculature.

Here we show the expression profile of members of the ZnT family in VSMCs and mouse aorta and identify ZnT3 and ZnT10 as important regulators of VSMC senescence. We show that Ang II, as well as zinc, downregulates ZnT3 and ZnT10 expression. This event decreases catalase expression, which leads to accumulation in ROS levels and the induction of senescence. Zinc regulates catalase protein stability by an ERK1/2-dependent mechanism. Zinc-induced senescence is prevented by the antioxidant N-acetyl-cysteine (NAC), suggesting that ZnT3 and ZnT10 work to prevent increases in ROS levels by modulating the expression of catalase.

## Materials and Methods

### Antibodies and DNA constructs

The following antibodies were used for western blots: Akt1/2 (H-136) was from Santa Cruz Biotechnology. p21 and P53 were from GeneTex. Rabbit polyclonal anti-catalase was from Calbiochem. Glutathione peroxidase 1 (GPx-1) was from Abcam. SOD1 was from Fisher Scientific. SOD2 was from Stressgen. Monoclonal antibodies against tubulin and β-actin and rabbit anti-PMP70 were from Sigma. Rabbit polyclonal antibodies against phospho-Akt (Ser 473), phospho-p38MAPK (Thr 180/Tyr 182) phospho-p44/42 MAPK (Thr 202/Tyr 204), p38MAPK and p44/42 MAPK were from Cell Signaling. Rabbit antibody against myc was from Bethyl Laboratories, Inc. Monoclonal antibodies against Tfr-R (H68.4) were from Invitrogen. Polyclonal antibodies against green fluorescent protein (GFP) were from Synaptic System (Göttingen, Germany). Monoclonal anti-GFP 3E6 and H_2_-dichlorofluorescin diacetate (H_2_DCFDA) were from Molecular Probes. Ang II and Dulbecco's modified Eagle's medium (DMEM) with 25 mM Hepes and 4.5 g/liter glucose were from Sigma. Human ZnT10 (NM_018713) myc-tagged plasmid was obtained from GeneCopoeia™ (Rockville MD). The human zinc transporters ZnT3-myc, ZnT3-GFP and ZnT4-myc were previously described [24]. ZnT5-GFP plasmids were obtained from Dr. Juan M. Falcon-Perez (CIC bioGUNE, Spain) [25]. FoxO1 *wt* and the constitutive active mutant FoxO1-CA were obtained from Dr. R. W. Alexander at Emory University.

### Cell culture and transfection

VSMCs from male Sprague-Dawley rat thoracic aortas, prepared using enzymatic digestion as described [26], were obtained from Dr. Kathy K. Griendling at Emory University. Cells were cultured until passage 12 in DMEM (Sigma) supplemented with 10% bovine serum (Invitrogen), 2 mM glutamine, 100 U/ml penicillin and 100 mg/ml streptomycin. HEK293T cells (ATCC N° CRL-1573) were cultured in DMEM medium (Cellgro, Herndon, VA; 4.5 g/l glucose) containing 10% FBS (Hyclone, Lolgan, UT), 100 U/ml penicillin and 100 mg/ml streptomycin. HEK293 cells transfected with ZnT10-myc plasmids were maintained in media containing 0.2 mg/ml G418 as described previously [27]. For Ang II treatment, cells were growth-arrested for 24 hrs in DMEM containing 0.5% bovine serum prior to the addition of 100 nM Ang II or zinc. VSMCs were incubated with 50 µM H_2_O_2_ (Sigma) for five days and media changed every 24 hrs. The ROS scavenger N-acetyl cysteine (NAC, Sigma, 1 mM), the Akt inhibitor V Tribicirine (TCN) and ERK1/2 inhibitor PD98059 were added to cells 30 min prior to the addition of 50 µM ZnSO_4_ and maintained during the zinc incubation.

For Transfection experiments, VSMCs at 70–90% confluency were transfected using the basic Nucleofector® (Lonza Walkersville, Inc.)) kit for primary smooth muscle cells (Lonza, p13 program). Cells were allowed to recover for 48 hrs before further treatments. HEK293 cells were transfected using 4 µl of Lipofectamine 2000 for 16 hrs. After 48 hrs, transfected cells were selected with 0.8 mg/ml G418 for one week. Colonies were isolated with cloning rings and expression of target proteins tested by immunofluorescence.

### Reverse transcription PCR (RT-PCR) and RNA interference

RNA was isolated from VSMCs grown in 60 mm petri dishes and from mouse aorta using the RNeasy kit (Qiagen), according to manufacturer's instructions. The SuperScript™ One Stept RT-PCR system (Invitrogen) was used with 0.5 µg of total RNA to amplify target genes. Primers used to determine the expression of SLC30A/ZnTs zinc transporters are described in Table S1. GAPDH control primers were designed to recognize both rat and mouse targets. Downregulation by siRNA of target genes was performed using the basic Nucleofector® kit for primary smooth muscle cells. Oligonucleotides used for siRNA are described in Table S2. Downregulation efficiency was determined by RT-PCR or by western blot as appropriate.

### Zinpyr-1 staining and immunofluorescence

Cells cultured in MatTek dishes (Mat Tek Corp) were treated with and without ZnSO_4_, TPEN or Ang II in DMEM, washed and incubated with 10 µM Zinpyr-1 (Ex/Em = 515/525) in DPBS for 30 min at 37°C. Cells were then washed in DPBS and imaged with a confocal microscope (Zeiss LSM 510 META) using the 488 nm argon laser line and filter set HQ480/40×;HQ535/50m (Chroma). Images were acquired using a Plan Apochromat 63× oil immersion objective NA = 1.4. Fluorescence intensity was determined using MetaMorph software 3.0 (Molecular Devices, Sunnyvale, CA).

Immunofluorescence was performed as described previously [28]. Briefly, cells grown on coverslips were fixed with 4% paraformaldehyde (PFA), washed with PBS and permeabilized in blocking buffer containing 0.02% saponin. Alexa Fluor® 488 and Alexa Fluor® 568 were used as secondary antibodies. Samples were examined using the 488- and 543-nm lines of the argon ion and green HeNe lasers with 515/30-nm band pass and 585-nm-long pass filters, respectively, in a confocal imaging system (Zeiss LSM 510 META).

### ROS measurements

VSMCs seeded in 24-well plates were growth-arrested for 24 hrs in DMEM and treated with or without 100 nM Ang II, ZnSO_4_ or TPEN for 30 min. After two washes with DPBS, cells were incubated with 10 µM 2′,7′-dichlorodihydrofluorescein diacetate (H_2_DCFDA) for 30 min, washed twice and fluorescence intensity determined using a CytoFluor multi-well plate reader (Applied Biosystems (ABI) PerSeptive Biosystems).

### NADPH oxidase activity

NADPH oxidase activity was measured in membrane fractions using lucigenin as described [29]. Briefly, VSMCs were incubated with or without 50 µM zinc for 4 hrs, washed and lysed in 20 mM KH_2_PO4 buffer, pH 7.4. Samples were sonicated and centrifuged for 15 min at 28,000×g. Pellets containing total membranes were resuspended in 50 mM KH_2_PO4 assay buffer and incubated with 0.1 mM lucigenin and 1 µM NADPH. NADPH oxidase activity was determined by subtracting the luminescence before and after the addition of the superoxide scavenger Tiron (100 mM).

### Senescence associated β-galactosidase staining

SA-β-gal activity was determined as described [5]. Cells plated at low density in 12-well plates were growth-arrested in DMEM containing 0.5% bovine serum for 24 hrs and incubated with Ang II, zinc, TPEN or H_2_O_2_ in the same media for 3 to 10 days. Cells were then washed twice with PBS, fixed with 0.2% glutaraldehyde in PBS for 10 min and incubated in 40 mM phosphate buffer containing 1 mg/ml X-Gal, 150 mM NaCl, 2 mM MgCl_2_, 5 mM K_3_Fe(CN)_6_ and 5 mM K_4_Fe(CN)_6_ overnight at 37°C. Cells were washed twice with PBS and kept in 70% glycerol. Images were acquired using an Olympus IX71 microscope using a 10× objective. SA-β-gal positive cells were counted in 10 different fields/well (3 to 6 individual wells per condition) and expressed as a percentage of total cell number. Detection of SA-β-gal with the Galacto-Light Plus™ System (Applied Biosystems) was performed according to manufacturer's instructions. After treatment, samples were lysed and luminescence determined after 20 min incubation using a TD-20/20 luminometer (Turner Designs, Sunnyvale CA).

### Western blots

VSMCs were lysed in lysis buffer (50 mM Hepes, pH 7.4, 50 mM NaCl, 1% Triton X-100, 5 mM EDTA, 10 mM sodium pyrophosphate, 50 mM sodium fluoride, and 1 mM sodium orthovanadate) plus anti-protease cocktail (Sigma) and total homogenates separated on 4–20% PAGE-SDS Criterion pre-cast gels (Bio Rad). Protein expression was determined by enhanced chemiluminescence using specific antibodies.

### Cell viability

Cells grown to 80% confluency in 24-well plates were incubated with ZnSO_4_ or TPEN in DMEM containing 0.5% bovine serum for one to ten days. Cell viability was determined by trypan-blue exclusion using a Neubauer chamber. 100% viability was determined in the absence of zinc or TPEN.

### Statistics

All data are depicted as average ± standard deviation of the mean. Statistical significance was determined by Student's t test and accepted at p<0.05(*). ** denotes p<0.01.

## Results

### Zinc increases ROS and induces senescence of VSMCs

**Figure 1:**
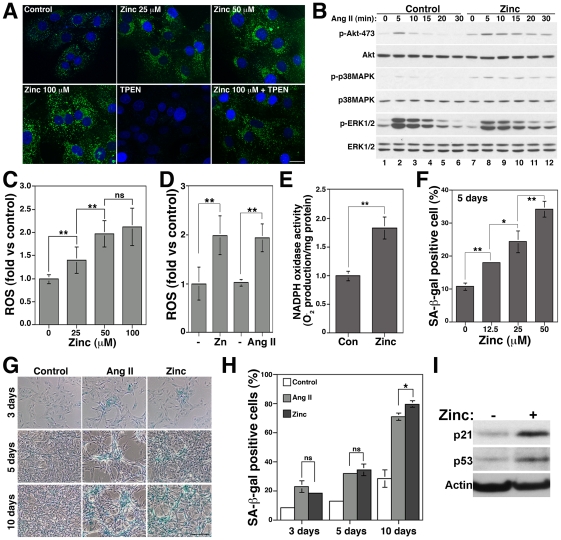
Zinc increases ROS levels, activates the ROS-dependent Ang II signaling pathway and induces senescence in VSMC. A) VSMCs incubated with 25, 50 or 100 µM zinc in the presence or absence of 25 µM TPEN for 1 hr were incubated with Zinpyr-1 and imaged by confocal microscopy (Bar = 20 µm). B) Cells pre-incubated with 100 µM zinc for 1 hr were incubated with 100 nM Ang II for 5 to 30 min, lysed and analyzed by immunoblot with the indicated antibodies. Cells treated with 25, 50 or 100 µM zinc (C) or with 50 µM zinc or 100 nM Ang II for 30 min (D) were incubated with H_2_DCFDA to determine ROS levels. E) NADPH oxidase activity was determined by lucigenin assay after 4 hrs with 50 µM zinc. F) SA-β-gal activity was determined after treatment with and without 12.5, 25 or 50 µM zinc for five days. G and H) Cells were treated with and without 100 nM Ang II or 50 µM zinc for three, five or ten days and senescence determined by counting SA-β-gal positive cells (Bar = 200 µm). I) Expression of p21 and p53 was determined after five days incubation with 50 µM zinc. * and ** denote p<0.05 and p<0.01, respectively. ns = non-significant differences.

To investigate if zinc homeostasis regulatory mechanisms and Ang II signaling pathways interact to promote vascular senescence, we first determined whether addition of exogenous zinc could increase intracellular zinc levels and enhance Ang II signaling in VSMCs. We incubated VSMCs with zinc and monitored the level of intracellular zinc using the specific zinc fluoroprobe Zinpyr-1 [30] (Fig. 1A). VSMCs show a basal vesicular and perinuclear staining that was increased by zinc and was reduced by the zinc chelator TPEN (Fig. 1A and Fig. S1A). We next tested if increases in intracellular zinc levels could affect Ang II signaling by monitoring Akt, p38MAPK and ERK1/2 phosphorylation (Fig. 1B). These three kinases mediate Ang II effects in VSMCs [31]. Pre-incubation with zinc increases Akt and p38MAPK and decreases ERK1/2 phosphorylation in basal conditions (Fig. 1B, lane 7) and after Ang II treatment (Fig. 1B, compare lanes 2–6 and 8–12). Since Akt and p38MAPK, but not ERK1/2, are activated by Ang II in a ROS-dependent manner in VSMCs [31] and senescence is induced by ROS [32], we tested whether zinc could increase ROS levels and induce senescence. Zinc increased ROS levels 1.4±0.28, 1.97±0.28 and 2.12±0.4-fold after incubations with 25, 50 and 100 µM zinc, respectively (n = 7, p<0.01) (Fig. 1C). Increases in ROS levels by 50 µM zinc (1.99±0.4-fold n = 8, p<0.01 vs control) were similar to the ones observed after Ang II treatment (1.94±0.28-fold n = 4, p<0.01 vs control) (Fig. 1D). Furthermore, zinc increased NADPH oxidase activity 1.82±0.18-fold (n = 3, p<0.01) (Fig. 1E).

To determine senescence, we incubated VSMCs with increasing concentrations of zinc for five days and measured SA-β-gal activity, a senescence marker. 50 µM zinc is the optimal concentration for long-term incubations based on cell viability assays (Fig. S1B) and ROS production (Fig. 1C). Senescence was increased from 10.8±1.1% to 18±0.34%, 24.4±3.36% and 34.33±2.42% after treatment with 12.5, 25 and 50 µM zinc, respectively (Fig. 1F). Similar to Ang II, zinc increased SA-β-gal staining over time (Fig. 1G and 1H). Zinc also increased the expression of the senescence markers p21 and p53 (Fig. 1I) as previously reported for Ang II in VSMCs [14]. Thus, zinc mimics Ang II by activating NADPH oxidase activity, increasing ROS levels, activating the ROS sensitive p38MAPK/Akt pathway and inducing senescence.

### Zinc is required for Ang II-induced senescence

**Figure 2:**
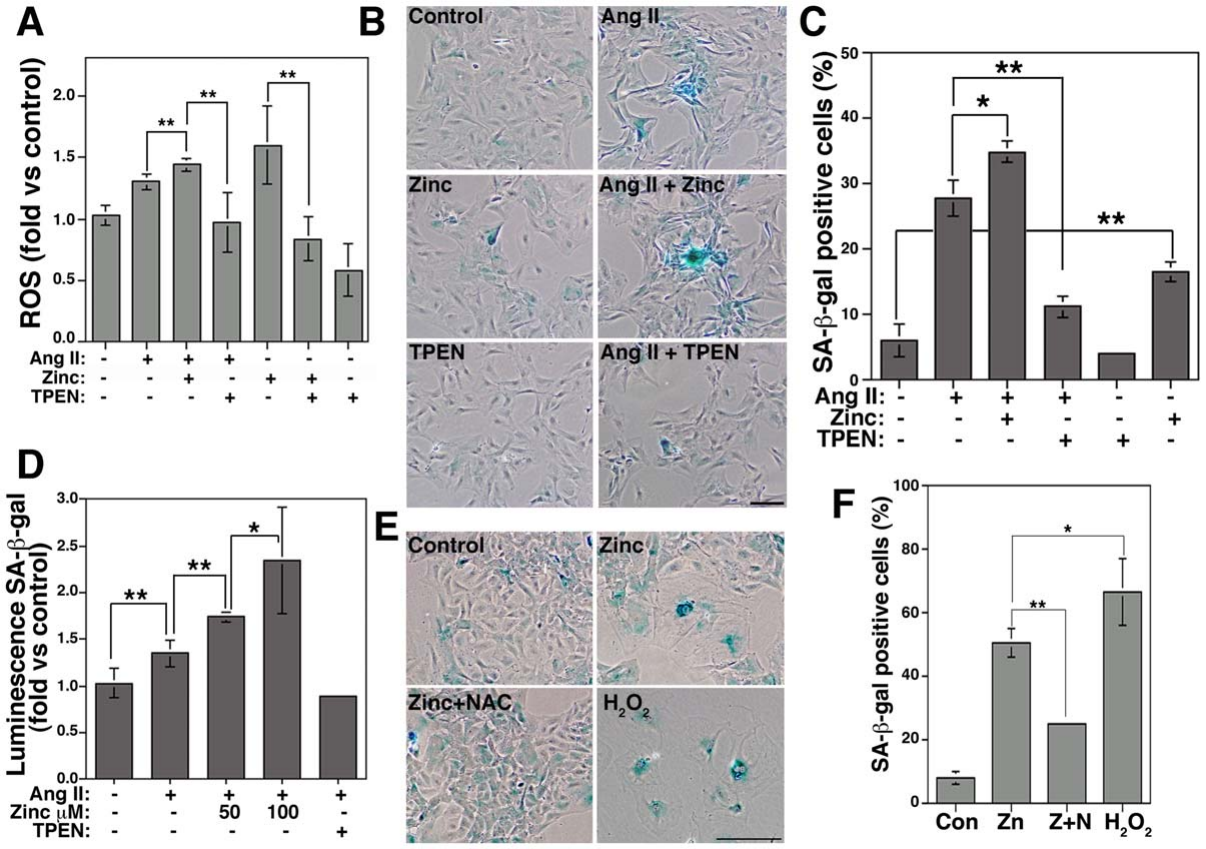
Zinc is required for Ang II-induced senescence by a ROS-dependent pathway. A) Cells incubated with and without Ang II in the presence or absence of 50 µM zinc or 100 nM TPEN for 30 min were incubated with H_2_DCFDA to determine ROS levels. SA-β-gal activity was determined after three (B–D) or five (E and F) days by counting SA-β-gal positive cells (C and F) or by quantitative luminescence (D). E and F) Cells were incubated with zinc, zinc plus 1 mM NAC or 50 µM H_2_O_2_ and senescence determined by SA-β-gal staining. Bar = 200 µm.

The fact that zinc mimics Ang II effects led us to hypothesize that zinc could be required for Ang II-induced senescence. This hypothesis predicts that Ang II-induced increases in ROS levels and senescence should be increased by zinc and prevented by TPEN (Fig. 2). ROS levels induced by Ang II were significantly increased by zinc (1.43±0.04-fold vs 1.3±0.06-fold, n = 6, p<0.01) and were reduced by TPEN (0.968±0.24-fold, n = 6, p<0.01). Further, basal as well as zinc-induced increases in ROS were also reduced by TPEN (Fig. 2A). Next, we incubated VSMCs with Ang II in the presence of zinc or TPEN for three days to determine senescence. Cell viability assays show that 100 nM TPEN is a non-toxic concentration for long-term incubations (Fig. S1C). This concentration is 100 times lower than the concentration (10 µM TPEN) described to remove zinc from zinc containing proteins such as transcriptional factors [33]. Zinc increases and TPEN reduces Ang II-induced senescence (Fig. 2B–D). Ang II treatment leads to 27.9±2.8% SA-β-gal positive cells compared to 6.1±2.4% in control cells (p<0.01). The effect of Ang II was increased to 34.9±1.6% (p<0.05 vs Ang II) when cells were co-incubated with 50 µM zinc, and was reduced to 11.3±1.65% (p<0.01 vs Ang II) when cells were co-incubated with 100 nM TPEN (Fig. 2C). Similar results were observed when SA-β-gal activity was determined by quantitative chemiluminescence using the Galacto-Light Plus™ System (Applied Biosystems) (Fig. 2D). We detected an increase of 1.35±0.14-fold (n = 6, p<0.01) in Ang II-induced senescence compared to control cells (1.03±0.16-fold). Incubation with 50 and 100 µM zinc increased Ang II-induced senescence 1.75±0.05-fold (n = 6, p<0.01) and 2.34±0.57-fold (n = 3, p<0.05), respectively, compared to Ang II alone. In contrast, TPEN prevents Ang II effects (0.88±0.03-folds, n = 3, p = 0.24 vs control). Thus, zinc increases ROS levels and senescence while TPEN prevents them. To further evaluate the dependence of ROS in zinc-induced senescence, we incubated VSMCs with zinc in the presence or absence of the antioxidant NAC (Fig. 2E and F). The increase in senescence induced by zinc (50.6±4.4%) was similar to incubation with H_2_O_2_ alone (66.9±10.44%, p<0.05 vs zinc). NAC significantly reduced zinc-induced senescence to 25±1.2% (p<0.01 vs zinc). All together, these data indicate that zinc is required for Ang II-induced senescence via a ROS-dependent mechanism.

### Ang II downregulates zinc transporters ZnT3 and ZnT10 to induce senescence

**Figure 3:**
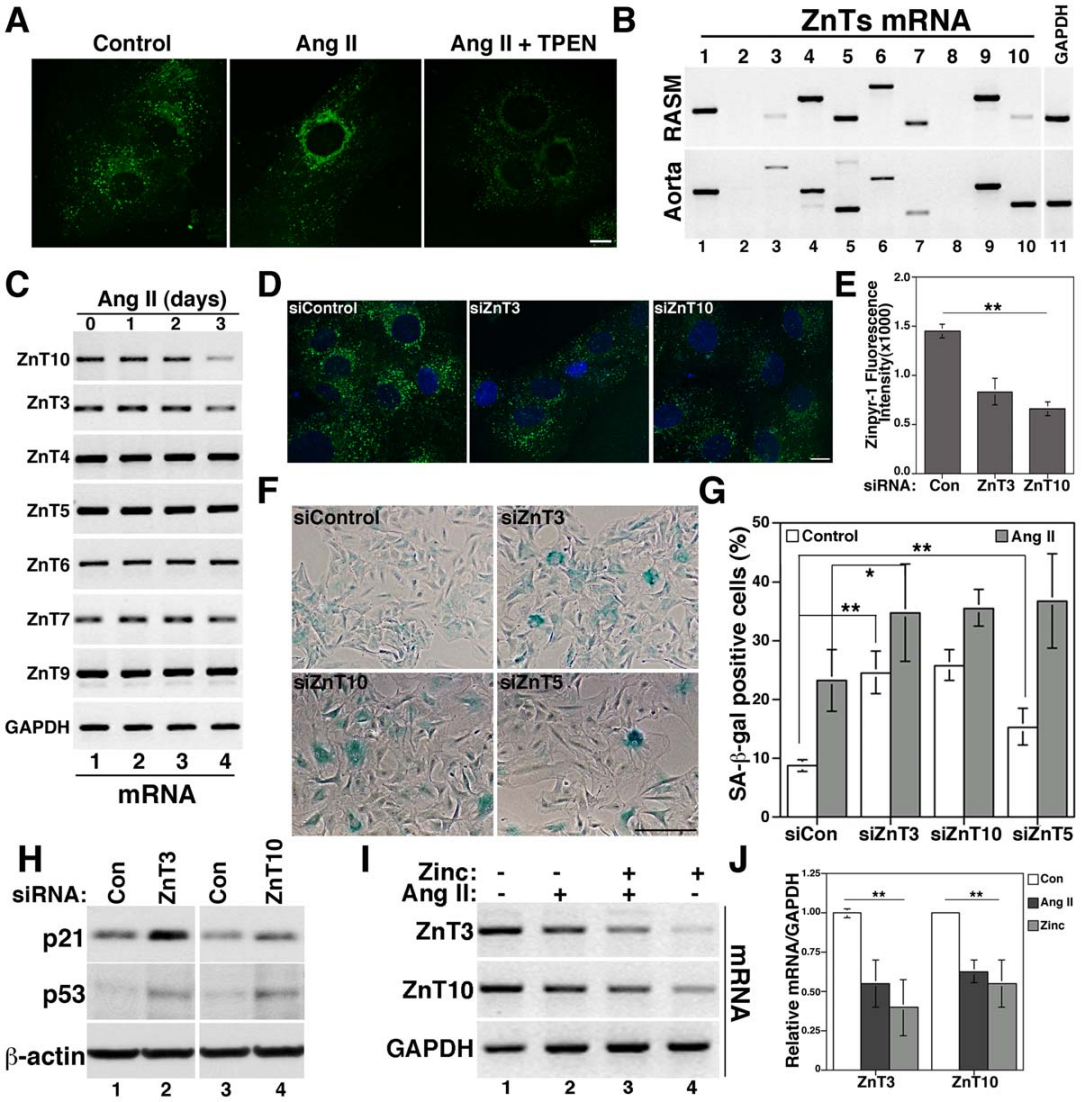
Ang II downregulates the zinc transporters ZnT3 and ZnT10 to induce senescence. A) VSMCs incubated with 100 nM Ang II for three days in the presence or absence of 100 nM TPEN were incubated with Zinpyr-1 and imaged by confocal microscopy. B) Total mRNA was used to determine ZnT1 to ZnT10 expression levels by RT-PCR in VSMCs and mouse aorta using GAPDH as control. All primers were designed to recognize mouse and rat ZnTs, except for ZnT1 and ZnT3 (see Table S1). C) mRNA prepared from VSMCs treated for one to three days with 100 nM Ang II was used to determine expression of the indicated ZnTs by RT-PCR. Cells treated with siRNA to downregulate ZnT3 and ZnT10 were imaged with Zinpyr-1 (D and E) or processed for SA-β-gal staining (F and G) or western blot to determine p21 and p53 expression (H). siZnT5-treated cells were analyzed for SA-β-gal staining (F and G). I and J) Cells were treated with Ang II, zinc or both for three days and samples analyzed by RT-PCR and quantified respect to GAPDH control. Bar = 10 µm in A and D and 200 µm in F. * and ** represent p<0.05 and p<0.01, respectively.

The observation that zinc is required for Ang II-induced senescence suggests that Ang II may affect the availability and/or distribution of free zinc and, therefore, zinc homeostasis to mediate senescence. To test this idea, we monitored changes in intracellular zinc using Zinpyr-1 staining in VSMCs treated for three days with Ang II alone or together with TPEN (Fig. 3A). Ang II induced changes in zinc distribution from a vesicular to a perinuclear Golgi-like staining in 64.3% of cells (14 different fields) compared to 21% (12 different fields) in control cells. This staining was reduced by TPEN. The change in zinc distribution suggests that Ang II may affect the expression of SLC30A/ZnT family members of zinc transporters, which work to reduce cytosolic zinc concentration by moving zinc out of the cells or inside vesicular compartments, such as endosomes and Golgi. We determined mRNA expression levels of SLC30A/ZnT family members by RT-PCR and found that VSMCs, as well as mouse aorta, express eight out of ten members of the ZnT family (Fig. 3B). We found ZnT1, that localizes at the plasma membrane [34], ZnT3 and ZnT4 that localize at endosomes [27], [35], and ZnT5, ZnT6 and ZnT7 found at the Golgi complex [36]. We also found ZnT9/HUEL, which was shown to translocate to the nucleus in a cell-cycle dependent manner [37], and ZnT10, whose subcellular localization and zinc transporter capacities have not been investigated. Next, we determined ZnTs mRNA expression levels in response to Ang II treatment. Ang II downregulated the expression of ZnT3 and ZnT10, but not other ZnTs (Fig. 3C and 3J).

To determine the functional consequences of ZnT3 and ZnT10 downregulation by Ang II, we knocked down their expression levels by siRNA (Fig. S2A and S2B) and determined senescence by SA-β-gal staining (Fig. 3F and 3G). ZnT3 and ZnT10 downregulation decreased the levels of vesicular zinc (Fig. 3D and 3E) and increased basal senescence to 24.72±3.6% and 25.97±2.65%, respectively. These levels are similar to those observed in cells treated with Ang II in control conditions (23.41±5.19%). Similar to Ang II and zinc treatments, downregulation of both ZnTs also increased the expression of the senescence marker p21 and p53 (Fig. 3H). Further, ZnT3 and ZnT10 downregulation increased Ang II-induced senescence (Fig. 3G). These data suggest that downregulation of ZnT3 and ZnT10 might lead to increases of zinc in the cytosol before the addition of Ang II, which could accelerate senescence (more SA-β-gal positive cells). This idea is supported by the fact that zinc alone increases, while TPEN prevents, senescence. This hypothesis predicts that increases of cytosolic zinc by downregulation of any other zinc transporter should also induce senescence. To test this idea, we knocked down the Ang II-insensitive ZnT5 by siRNA (Fig. S2C) and found that this treatment also induces senescence (Fig. 3F and 3G). siZnT5 increased basal (15.48%±3.05%, p<0.01 vs control cells) as well as Ang II-induced senescence (36.88±7.9%, p<0.01, vs Ang II in control cells), suggesting that zinc homeostasis dysfunction mediates senescence of VSMCs. Further, no changes in cellular distribution of Ang II-insensitive ZnTs, such as ZnT5 and ZnT4 was observed by Ang II (Fig. S3).

Since expression of zinc transporters such as ZnT1 are transcriptionally regulated by zinc [38] and zinc induces senescence (Fig. 1F–I), we asked whether ZnT3 and ZnT10 could be downregulated by zinc. Both ZnTs mRNA were downregulated by zinc similar to Ang II (Fig. 3I and 3J), suggesting that increase in zinc levels could be mediated by Ang II in an early event upstream of ZnTs downregulation. All together, these data show that Ang II downregulates the zinc transporters ZnT3 and ZnT10 to induce senescence.

### Overexpression of ZnT3 and ZnT10 prevents Ang II-induced senescence

**Figure 4:**
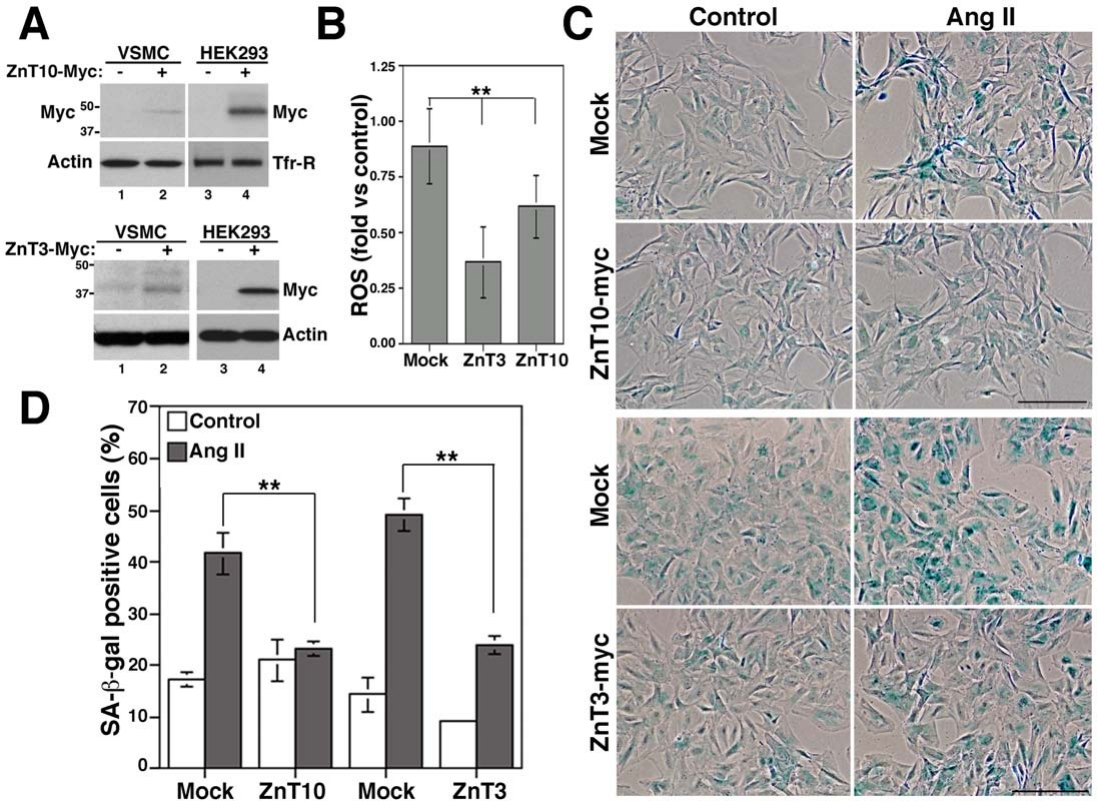
Overexpression of ZnT3 and ZnT10 decreases ROS and prevents Ang II-induced senescence. A) ZnT3 and ZnT10 expression was determined by immunoblot with anti-myc antibodies in control and transfected VSMCs and HEK293 cells, using β-actin and transferrin receptor (Tfr-R) as loading controls. B) ROS levels were measured with H_2_DCFDA in mock or ZnT3 and ZnT10 transfected VSMCs cells. C and D) Senescence was determined by counting SA-β-gal positive cells in control and ZnT3-myc and ZnT10-myc transfected cells treated with 100 nM Ang II for five days. Bar = 200 µm in C. ** represents p<0.01.

To further test the role of ZnT3 and ZnT10 in Ang II-induced senescence, we asked whether overexpression of these transporters could prevent Ang II effects. Western blots with anti-myc antibodies confirmed ZnT10 and ZnT3 overexpression and show that VSMCs express lower levels of these transporters compared with HEK293 cells after transfection (Fig. 4A). A discrete overexpression of ZnT3 or ZnT10 decreased ROS levels 0.37±1.6 and 0.62±0.14-fold, respectively (Fig. 4B) and prevented Ang II-induced senescence (Fig. 4C and D). VSMCs overexpressing ZnT10-myc were 23.3±1.35% positive for SA-β-gal compared to 41.6±4%, p<0.05 in mock-transfected cells treated with Ang II. Non-significant (ns) differences were observed between transfected and mock-transfected cells in basal conditions or between transfected cells with and without Ang II (23.3±1.4 and 21±4%, respectively, p = 0.4). Similarly, ZnT3 overexpression reduced Ang II-induced senescence from 49.23±3.08% in mock-transfected cells to 23.91±1.68% (p<0.01). Thus, overexpression of ZnT3 and ZnT10 mimics the effects of TPEN by decreasing ROS levels and preventing senescence, suggesting that senescence is mediated by zinc. Thus, these data show that Ang II-induced senescence is a zinc-dependent process mediated by the downregulation of ZnT3 and ZnT10.

### Characterization of ZnT10 localization and zinc transport capacity

**Figure 5:**
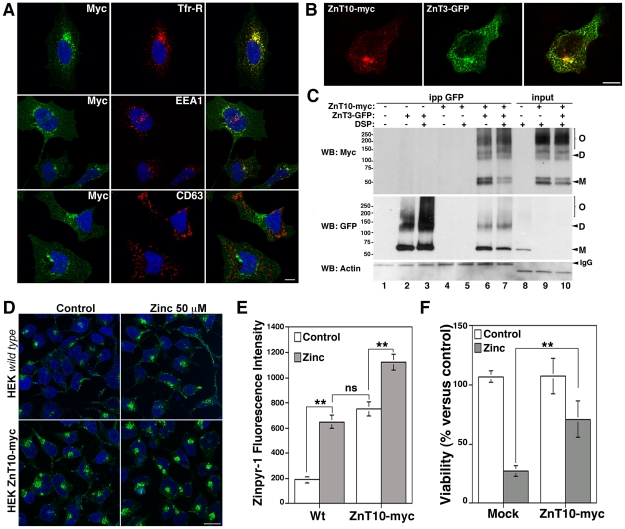
ZnT10 localizes and transports zinc into early/recycling endosomes in HEK293 cells. HEK293 cells transfected with ZnT10-myc alone (A) or together with ZnT3-GFP (B) were fixed in 4% PFA and analyzed by immunofluorescence with monoclonal antibodies against Tfr-R, EEA1 and CD63 (A) and polyclonal antibodies against myc (A and B). Images were acquired with a confocal microscope using a 63×/oil objective. C) HEK293 cells transiently transfected with ZnT10-myc and ZnT3-GFP were cross-linked with 1 mM DSP, lysed and Triton X-100 soluble extracts immunoprecipitated with monoclonal antibodies against myc. ZnT3 and ZnT10 interaction (lanes 6 and 7) was determined by western blots using polyclonal antibodies against myc and GFP. 10 µg of Triton X-100 soluble extracts were used as inputs (lanes 8–10). D and E) Control and HEK293 stable cells lines expressing ZnT10-myc were incubated with and without zinc and stained with Zinpyr-1. Fluorescence intensity was determined by confocal microscopy and quantified using MetaMorph software. F) Cell viability was determined by trypan-blue exclusion in HEK293 control and ZnT10-myc expressing cells after incubations with 100 µM zinc for 24 hrs. Bar = 10 µm in A, B and D.

We hypothesize that overexpression of ZnT3 or ZnT10 should prevent increases in cytosolic zinc to prevent senescence. However, ZnT10 zinc transport capacities have not been explored. The transport capacity of ZnT3 is well-documented [24], [27]. To investigate the mechanism by which ZnT10 downregulation induces senescence, we first determined ZnT10 subcellular localization and zinc transport capacities. We analyzed ZnT10 subcellular localization in HEK293 cells transfected with the human ZnT10 myc tagged (Fig. 5A). We choose HEK293 cells because they are easily transfected (Fig. 4A) and suitable for morphological studies. In this cell line, ZnT10 strongly colocalizes with markers of recycling endosomes, such as the transferrin receptor (Tfr-R) (69.39±11.21%, n = 14 cells) and rab11 (data not shown) and with the early endosome marker EEA1 (16.21±4.38%, n = 13 cells), but not with syntaxin 8 (data not shown) or CD63 (1.83±0.93%, n = 10 cells), markers of late endosome and lysosomes, respectively. Moreover, ZnT10 co-localizes (63.7±12%, n = 12 cell, Fig. 5B) and interacts (Fig. 5C) with ZnT3 which also resides in Tfr-R-positive endosomes [27]. Immunoprecipitation with anti-GFP antibodies brings down ZnT10-myc in cells co-expressing ZnT10-myc and ZnT3-GFP in the presence or absence of the cross-linker DSP (Fig. 5C, lanes 6 and 7). We previously used DSP to detect labile interactions and to demonstrate the formation of ZnT3 dimers [24]. ZnT10 and ZnT3 form stable dimers (D) and oligomers (O) that may mediate zinc accumulation/transport into recycling endosomes.

We next determined ZnT10 zinc transport capacities in colonies of HEK293 cells permanently transfected with ZnT10-myc (Fig. S4, colony N° 18, 100% expression), using Zinpyr-1 to visualize vesicular zinc accumulation (Fig. 5D and 5E). Fluorescence intensity (zinc accumulation) was higher in ZnT10-transfected compared with mock-transfected cells in both basal (751.43±56.4 vs 190.46±23.12, n = 39 cells) and zinc-treated conditions (1,121±61 vs 650±54.7, n = 37 cells). Zinpyr-1 fluorescence staining resembles the vesicular and perinuclear ZnT10-positive signal detected with anti myc antibodies by immunofluorescence (Fig. S4). To further assess ZnT10 transport capacities, we determined whether ZnT10 overexpression could confer resistance to zinc toxicity (Fig. 5F). Cells overexpressing ZnT10-myc showed a significant increase in cell viability (71±15%, n = 6, p<0.01) compared with mock-transfected cells (27.5±4.6%) after a challenge with toxic levels of zinc. Thus, ZnT10 functions to transport zinc into early/recycling endosomes.

### Zinc and ZnT3/ZnT10 downregulation decrease catalase expression to induce senescence

**Figure 6:**
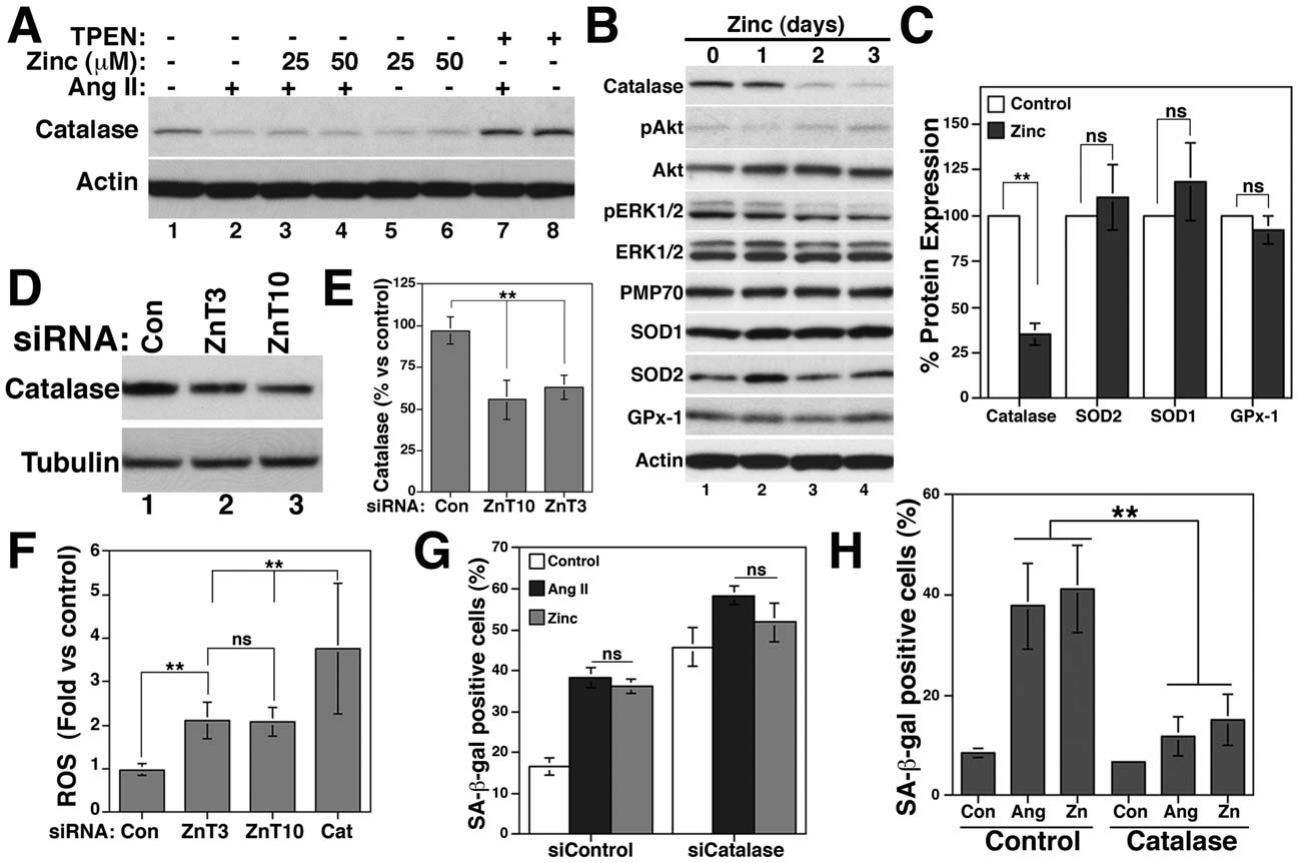
Downregulation of catalase by zinc, Ang II, siZnT3 and siZnT10 mediates senescence in VSMCs. A) Cells incubated with and without 100 nM Ang II, zinc (25 or 50 µM) or 100 nM TPEN for five days were lysed and catalase expression determined by western blots. B and C) VSMCs incubated with and without 50 µM zinc for one, two or three days were lysed and protein expression of the indicated antioxidant enzymes determined by western blots and quantified after 3 days (C). D and E) Catalase expression was determined by western blot after ZnT3 and ZnT10 knock down by siRNA. F) Cells treated with siRNA to downregulate ZnT3, ZnT10 or catalase (Cat) were incubated with H_2_DCFDA to determine ROS levels. SA-β-gal positive cells were counted in siControl and siCatalase-treated cells (G) and in catalase-transfected cells (H) after five days treatment with and without Ang II or zinc 50 µM. ** and ns denote p<0.01 and non-statistic differences, respectively.

We next explored the mechanism by which zinc and ZnT3/ZnT10 downregulation promotes senescence. We focused on catalase because transient activation of the ROS-dependent Akt, but not the ERK1/2 pathway, mediates catalase downregulation by Ang II in VSMCs [39]. We show that zinc increases ROS levels (Fig. 1C and 1D) and activates Akt, but not ERK1/2 (Fig. 1B). Zinc reduced catalase protein expression (Fig. 6A and Fig. S5A), similar to Ang II alone (Fig. 6A, 47±4.94%, n = 5, p<0.01 vs control). In contrast, chelation of zinc with TPEN increases basal levels of catalase and prevents its downregulation by Ang II (Fig. 6A and Fig. S5A). These data support the idea that TPEN prevents Ang II-induced senescence by preventing increases in ROS levels (Fig. 2A) and catalase downregulation. Time course experiments show that catalase, but not the peroxisomal membrane protein PMP70, was significantly downregulated after two days of zinc incubation (Fig. 6B and 6C), indicating that peroxisome biogenesis was not affected. No significant changes were observed in other antioxidant enzymes, such as SOD1, SOD2 or glutathione peroxidase-1 (GPx-1) after three days of zinc incubation (Fig. 6B and 6C). Akt and ERK1/2 phosphorylation was increased and decreased by zinc, respectively (Fig. 6B). Since zinc also downregulates ZnT3 and ZnT10, we asked whether downregulation of zinc transporters is upstream of catalase reduction. Knock down of ZnT3 and ZnT10 by siRNA decreased catalase (Fig. 6D and 6E) and increased ROS levels 2.11±0.43-fold and 2.07±0.33-fold (n = 12, p<0.01) compared to siControl-treated cells (0.98±0.14), respectively (Fig. 6F). Downregulation of catalase increased ROS levels 3.75±1.51-fold (n = 9, p<0.01).

To determine the role of catalase in the senescence mechanism induced by zinc and ZnT3/ZnT10 downregulation, we knocked down catalase by siRNA and determined senescence (Fig. 6G). p21 expression (Fig. S5B) as well as SA-β-gal staining was increased by siCatalase (45.81±4.79%) compared to siControl cells (16.64±2.03%, p<0.01). Ang II (38.36±2.46%) and zinc-induced (36.24±1.9%) senescence was increased to 58.4±2.39% and 51.81±4.65%, respectively by catalase siRNA (Fig. 6G). Consistently, overexpression of catalase (Fig. S5C) significantly reduced both Ang II (37.18±8.6%) and zinc-induced (45.1±8.7%) senescence to 11.8±3.8% and 15±5.1%, respectively (Fig. 6H).

### Zinc downregulates catalase by a post-transcriptional ERK1/2-dependent mechanism

**Figure 7:**
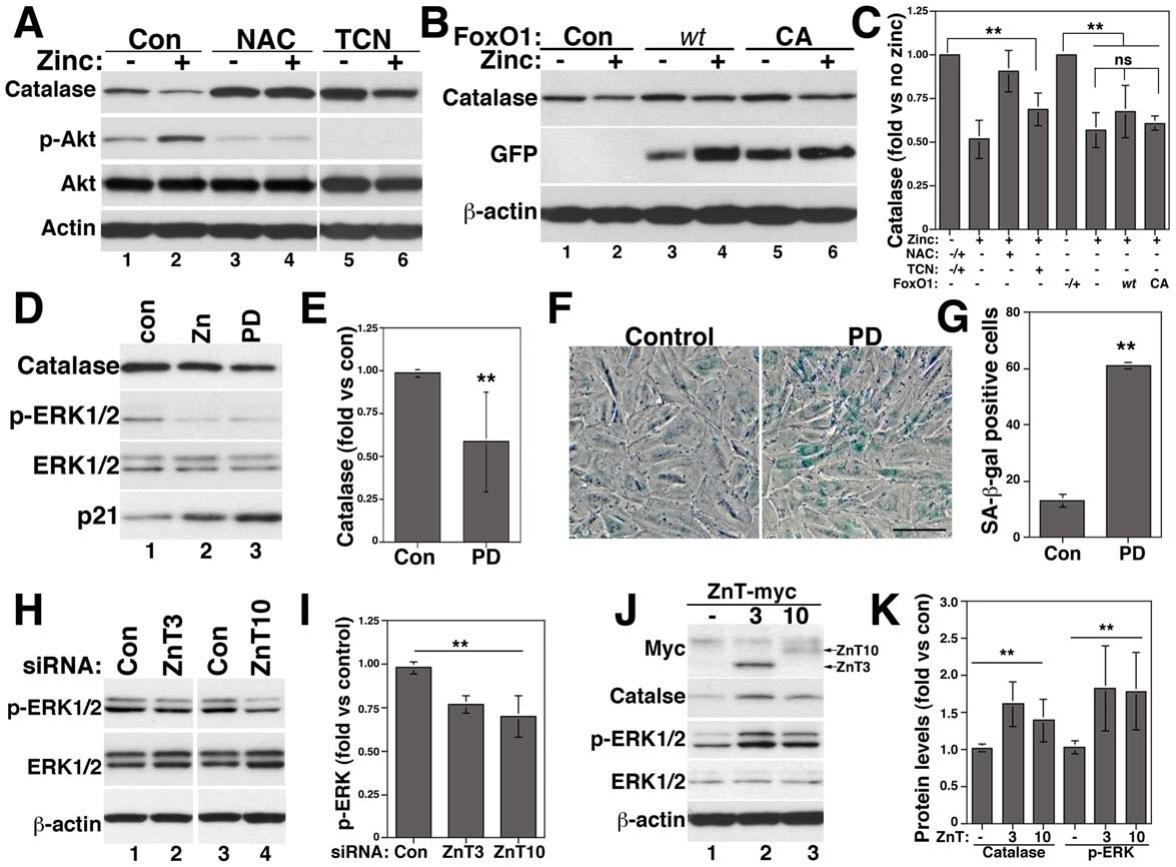
Catalase is downregulated by zinc by an Akt-independent ERK1/2-dependent post-transcriptional pathway. VSMCs treated with and without 1 mM NAC, 1 µM of the Akt inhibitor V tricibirine (TCN) (A and C) or transfected with FoxO1 *wt* or FoxO1-CA (B and C) were incubated with or without 50 µM zinc for three days and protein expression determined by western blots with the indicated antibodies. C) The effect of zinc was quantified for each condition independently, considering basal levels of catalase with or without NAC, TCN or FoxO1 overexpression as 1. Cells incubated for three days with 50 µM zinc or 2 µM of the ERK1/2 inhibitor PD98059 (PD) were processed for western blot to determine catalase expression (D and E) or for SA-β-gal staining (F and G). Bar = 100 µm in F. VSMCs treated with siZnT3 and siZnT10 (H and I) or transfected with ZnT3-myc or ZnT10-myc plasmids (J and K) were lysed and protein levels of p-ERK1/2 (I and K) and catalase (K) quantified by western blots. ** and ns denote p<0.01 and non-statistic differences, respectively.

Ang II downregulates catalase by an Akt/FoxO1-dependent and ERK1/2-independent transcriptional mechanism [39]. Zinc increases Akt phosphorylation, suggesting that zinc may act by a similar mechanism. In fact, NAC that prevented zinc-induced senescence (Fig. 2F) also prevented Akt phosphorylation and catalase downregulation by zinc (Fig. 7A and 7C). However, inhibition of Akt with the inhibitor V tricibirine (TCN) increased basal levels of catalase similar to NAC, but failed to prevent catalase downregulation by zinc (Fig. 7A, lanes 5 and 6 and 7C). No changes in Akt expression were observed by NAC or TCN. To further test the role of the Akt/FoxO1 pathway on zinc effects, we overexpressed FoxO1 *wild type* and the constitutive active mutant FoxO1-CA (T24A/S319A) that is not phosphorylated and inactivated by Akt [40]. Overexpression of FoxO1 constructs increased basal levels of catalase (Fig. 7B, lanes 3 and 5). Zinc downregulated catalase expression similarly in control conditions and after FoxO1 *wt* and CA overexpression (Fig. 7B and 7C), suggesting that zinc might regulate catalase by a post-transcriptional mechanism. Consistent with this idea, catalase mRNA levels were not affected by zinc (Fig. S6A and B).

We next tested whether inhibition of ERK1/2 by zinc could mediate zinc effects. Similar to zinc, the ERK1/2 inhibitor PD98059 (PD) decreased catalase and increased p21 expression (Fig. 7D and 7E) and SA-β-gal staining (Fig. 7F and 7G). ZnT3 and ZnT10 siRNA also decreased ERK1/2 phosphorylation (Fig. 7H and 7I) while ZnT3 and ZnT10 overexpression increased ERK1/2 phosphorylation and catalase expression (Fig. 7J and 7K). Thus, zinc and ZnT3/ZnT10 regulate catalase expression by an Akt-independent ERK1/2-dependent post-transcriptional mechanism.

All together, these data demonstrate that zinc and downregulation of ZnT3 and ZnT10 decrease catalase expression leading to increases of ROS levels senescence of VSMCs.

## Discussion

**Figure 8:**
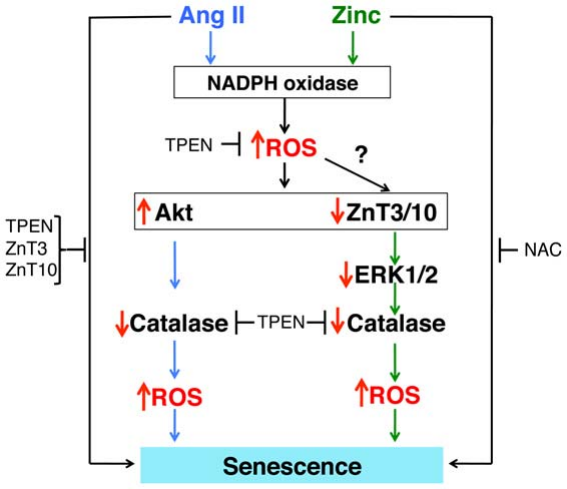
Model representing Ang II and zinc-dependent senescence mechanisms. Similar to Ang II, zinc activates NADPH oxidase activity, increases ROS levels and Akt phosphorylation and downregulates ZnT3, ZnT10 and catalase. However, Ang II-induced downregulation of catalase is mediated by an Akt-dependent transcriptional mechanism, while zinc-induced downregulation of catalase is mediated by an ERK1/2-dependent post-transcriptional mechanism. Downregulation of catalase further increases ROS that mediates senescence. Perpendicular red arrows indicate increased or reduced expression.

Our findings, summarized in Fig. 8, demonstrate that Ang II-induced senescence requires a zinc-dependent pathway mediated by the downregulation of the zinc transporters ZnT3 and ZnT10. This event would disrupt zinc homeostasis leading to downregulation of catalase and increases in ROS levels that promotes senescence. Zinc shows similar effects to Ang II. Zinc increases ROS levels, activates NADPH oxidase activity (Fig. 1C–E), decreases ZnT3, ZnT10 (Figs. 3I an J) and catalase expression (Fig. 6A–C) and increases senescence (Fig. 1G–I). In contrast, the zinc chelator TPEN, as well as overexpression of ZnT3 or ZnT10, decrease ROS levels (Fig. 2A and 4B), increase catalase expression (Figs. 6A, 7J and 7K) and prevent Ang II-induced senescence (Figs. 2B–D, 4C and 4D). Consistently, downregulation of ZnT3, ZnT10 or catalase by siRNA increases ROS (Fig. 6F) and senescence (Figs. 3F, 3G and 6G). Further, zinc-induced downregulation of catalase and senescence is prevented by NAC (Figs. 7A, 2E and 2F). To our knowledge, this is the first evidence linking Ang II signaling and zinc homeostasis regulatory mechanisms, such as zinc transporters, in a senescence pathway.

The observations that zinc increases ROS levels and activates Akt, similar to Ang II, lead us to speculate that zinc levels could increase after Ang II stimulation. Increases in zinc levels could be caused by release of zinc from zinc containing proteins and/or from membrane compartments. In VSMCs, we identified eight out of the ten members of the SLC30A/ZnT family of zinc transporters, which are responsible for zinc accumulation in membrane compartments. ZnT3, ZnT4 and ZnT10 localize in endosomes and ZnT5, ZnT6 and ZnT7 in the Golgi complex. Thus, endosomes and/or Golgi could serve as reservoirs of free zinc for signal transduction in VSMCs. Alternatively, zinc could be ejected from zinc-binding proteins, such as MTs. MT is a family of cysteine-rich metal-binding proteins that bind labile zinc, which could be released by cysteine oxidation [41]. Zinc is redox inert but it binds to proteins through sulfurs of cysteines, allowing its rapid exchange by the redox environment. An interesting possibility is that Ang II-induced increases of ROS levels could lead to zinc release from proteins/compartments leading to increases in free zinc. This free zinc could further activate NADPH oxidase activity and increase ROS leading to the induction of senescence. The source of zinc that activates Ang II signaling and increases ROS remains to be elucidated.

Our data are in agreement with the reported effects of zinc in signaling pathways in other cellular systems. In HepG2 human hepatoma cells, zinc activates the PI3K/Akt pathway, inducing the phosphorylation, inactivation and nuclear exclusion of FoxO1 [42]. Zinc shows insulin-mimetic effects by activating the insulin-dependent signaling cascade [43]. The effects of zinc in the insulin pathway are ROS-dependent. In rat adipocytes, zinc increases the generation of H_2_O_2_ and superoxide to stimulate glucose metabolism [44]. Moreover, zinc activates NADPH oxidase activity and increases ROS in cortical neurons [45], [46] and in the renal epithelial cell line, LLC-PK1 [47]. Here we show that zinc also activates a NADPH oxidase in VSMCs (Fig. 1E). Aortic VSMCs express Nox1 and Nox4 [48], which has been associated with cellular senescence [49]. The nature of the zinc-induced Nox(es) remains to be elucidated.

Zinc and Ang II regulate catalase expression by different mechanisms. Ang II downregulates catalase by an Akt-dependent (ERK1/2-independent) acetylation/inactivation of the transcriptional coactivator PGC-1α (peroxisome proliferator-activated receptor gamma co-activator-1α), leading to reduced binding of FoxO1 to the catalase promoter [39]. Akt and its upstream kinase p38MAPK, but not ERK1/2, are activated by Ang II in a ROS-dependent manner in VSMCs [31]. Although zinc activates Akt phosphorylation, Akt inhibition, as well as overexpression of FoxO1 *wt* or a constitutive active mutant (FoxO1-CA), failed to prevent zinc-induced downregulation of catalase. Instead, zinc decreases catalase by decreasing ERK1/2 phosphorylation without affecting catalase mRNA levels (Fig. S6A and B). Inhibition of ERK1/2 phosphorylation with PD98059 decreases catalase expression and induces senescence (Fig. 7D–G). siZnT3/ZnT10 and ZnT3/ZnT10 overexpression decreases and increases ERK1/2 phosphorylation and catalase, respectively (Fig. 7H–K), suggesting that ZnT3/ZnT10 are upstream of ERK1/2. These data are in agreement with recent observations that ZnT3 regulates presynaptic ERK1/2 signaling, which is required for hippocampus-dependent memory [50]. The data presented here have important implications in the possible role of ZnT3 regulating ROS levels in the brain. Future studies will determine whether regulation of ROS levels by ZnT3/ZnT10 is also observed in other tissues.

The differential mechanism of catalase downregulation by Ang II and zinc may explain why zinc and siZnT3/siZnT10 show additive effects on Ang II-induced senescence. At least two pathways are involved in catalase downregulation. One involves a transcriptional mechanism mediated by Ang II-dependent activation of a ROS/Akt/FoxO1 pathway [39]. The anti-oxidant NAC, which inhibits Akt phosphorylation as well as the Akt inhibitor TCN, increase basal levels of catalase (Fig. 7A). Consistently, overexpression of FoxO1 *wt* and the Akt-insensitive mutant FoxO1-CA also increase catalase expression (Fig. 7B), suggesting that catalase is in fact regulated by a ROS/Akt/FoxO1 transcriptional mechanism. The second is a zinc-dependent post-transcriptional mechanism involving ROS/ZnT3/ZnT10/ERK1/2 that regulates catalase protein stability. Ang II also downregulates ZnT3 and ZnT10, but activates ERK1/2 signaling. Addition of zinc to Ang II-treated cells will decrease ERK1/2 signaling leading to a decrease in catalase mRNA levels and protein stability, which will further increase senescence. Zinc may affect catalase protein stability by preventing synthesis and/or increasing degradation. For example, zinc was shown to increase the ubiquitination of the phosphatase PTEN leading to its proteasome-dependent degradation with the subsequent activation of the PI3K/Akt pathway [51]. Catalase is also regulated by ubiquitination and proteosome degradation in mouse embryo fibroblasts and HEK293 cells [52].

Here, we reported the subcellular localization and zinc transport capacities of the zinc transporter ZnT10. ZnT10 localizes in rab11 and Tfr-R positive recycling endosomes, similarly to the localization of ZnT3 in PC12 cells [27]. Using Zinpyr-1 staining and zinc toxicity assays, we show that ZnT10 indeed transports zinc likely into early/recycling endosomes. We hypothesize that downregulation of ZnT3 or ZnT10 would disrupt zinc homeostasis by increasing its concentration in the cytosol. This hypothesis predicts that downregulation of any other ZnT would induce similar phenotypes. In fact, we found that ZnT5 siRNA, a zinc transporter localized in the Golgi complex [36] and that is insensitive to downregulation by Ang II, induces basal senescence. siZnT5 also increases Ang II-induced senescence to similar levels compared with siZnT3 and siZnT10 (Fig. 3F and G). These data suggest that ZnT3, ZnT10 and ZnT5 regulate zinc homeostasis to prevent increases of zinc in the cytosol and to prevent senescence.

In conclusion, we demonstrated that Ang II-induced senescence is a zinc-dependent process regulated by the downregulation of ZnT3 and ZnT10 leading to the decreased expression of catalase. Zinc mimics Ang II by increasing ROS levels, activating NADPH oxidase activity and Akt, decreasing ZnT3 and ZnT10, reducing catalase and inducing senescence. Our studies suggest that zinc may also affect other Ang II-induced and/or ROS-dependent processes, such as hypertrophy and migration of smooth muscle cells. These findings inform a cellular and molecular mechanism to understand the putative role of zinc homeostasis dysfunction in the development of atherosclerosis. We propose that manipulation of metal pools may modify the outcome of cardiovascular diseases. Future experiments will determine if zinc homeostasis dysfunction, such as zinc deficiencies in vivo, may exacerbate Ang II maladaptive effects. This information will provide important insight into possible nutritional interventions to lessen Ang II-induced age-dependent diseases.

## Supporting Information

Figure S1 **Intracellular zinc measurements and cell viability in response to long-term incubations with zinc or TPEN.** VSMCs were incubated with and without zinc (25 to 100 µM) or TPEN (100 or 250 nM) for 1 hr (A) or for one to ten days (B and C). A) Quantification of Zinpyr-1 fluorescence intensity shown in Fig. 1A was determined using MetaMorph software and expressed as mean ± SE. B and C) Cell viability was determined by trypan-blue exclusion. **: p<0.01, ns = non-significant differences.
(TIF)

Figure S2 **Expression of ZnT3, ZnT5 and ZnT10 decrease after siRNA treatment.** Cells were incubated with siRNAs to downregulate ZnT3 (A), ZnT10 (B) or ZnT5 (C). Samples were separated in agarore gels and images inverted using Photoshop software. Relative mRNA levels were calculated with respect to GAPDH expression. **: p<0.01.
(TIF)

Figure S3 **Subcellular localization of ZnT4 and ZnT5 is not affected by Ang II in VSMCs.** Cells transfected with ZnT4-myc or ZnT5-GFP plasmids were incubated with or without Ang II for three days. Cells were fixed and incubated with anti-myc or anti-GFP antibodies. Images were acquired using a confocal microscope. Bar = 10 µm.
(TIF)

Figure S4 **Expression of ZnT10-myc in transfected HEK293 cells.** Permanently transfected HEK293 cells (colony N°18) were fixed with 4% PFA and incubated with polyclonal anti-myc antibodies. Alexa Fluor® 568 was used as a secondary antibody. Samples were imaged using a confocal microscope. Bar = 10 µm.
(TIF)

Figure S5 **Catalase expression is downregulated by zinc and siCatalase.** VSMCs were incubated with 50 µM zinc or 100 nM TPEN for five days (A), siCatalase (B) or transfected with plasmids containing catalase (C). Data on A represent quantification of catalase expression shown in Fig. 6A, expressed as percent versus control. **: p<0.01.
(TIF)

Figure S6 **Zinc downregulates catalase by a post-transcriptional mechanism.** A) Catalase mRNA levels were determined by RT-PCR after treatment with or without 50 µM zinc for three days. B) Catalase relative mRNA was calculated respect to GAPDH expression.
(TIF)

Table S1 **Primers used to determine mRNA levels of mouse (m) and rat (r) ZnTs by RT-PCR.** Primers were design to recognize mouse and rat ZnTs, except for ZnT1 and ZnT3.
(TIF)

Table S2 **Oligonucleotide sequence for siRNA downregulation.**
(TIF)
