# Supplementary figures and images for: Angiotensin II Requires Zinc and Downregulation of the Zinc Transporters ZnT3 and ZnT10 to Induce Senescence of Vascular Smooth Muscle Cells
Source: PLoS One. 2012 Mar 12;7(3):e33211. doi: 10.1371/journal.pone.0033211 (PMC3299759; doi:10.1371/journal.pone.0033211)

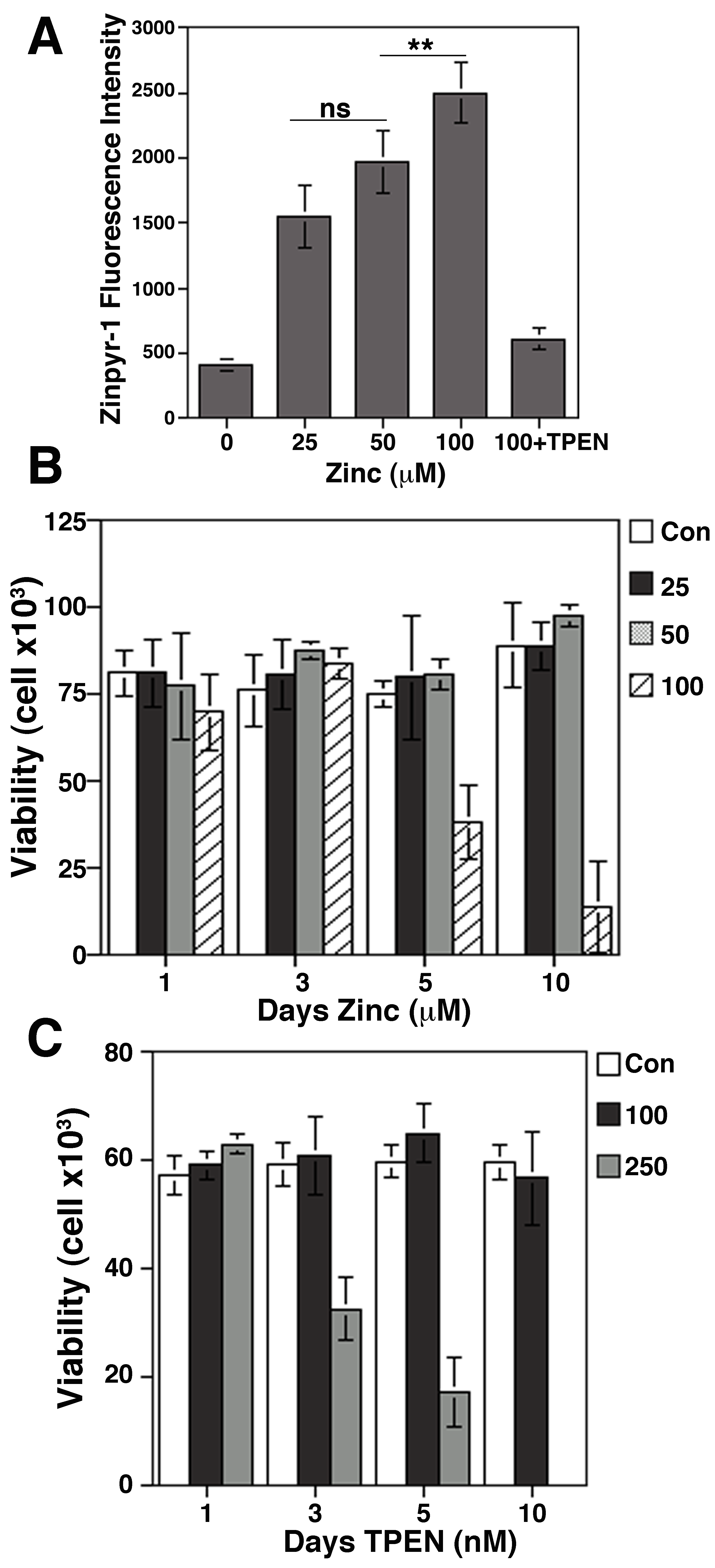

Supplement: Figure S1 — Intracellular zinc measurements and cell viability in response to long-term incubations with zinc or TPEN. VSMCs were incubated with and without zinc (25 to 100 µM) or TPEN (100 or 250 nM) for 1 hr (A) or for one to ten days (B and C). A) Quantification of Zinpyr-1 fluorescence intensity shown in Fig. 1A was determined using MetaMorph software and expressed as mean ± SE. B and C) Cell viability was determined by trypan-blue exclusion. **: p<0.01, ns = non-significant differences. (TIF) [file pone.0033211.s001.tif]

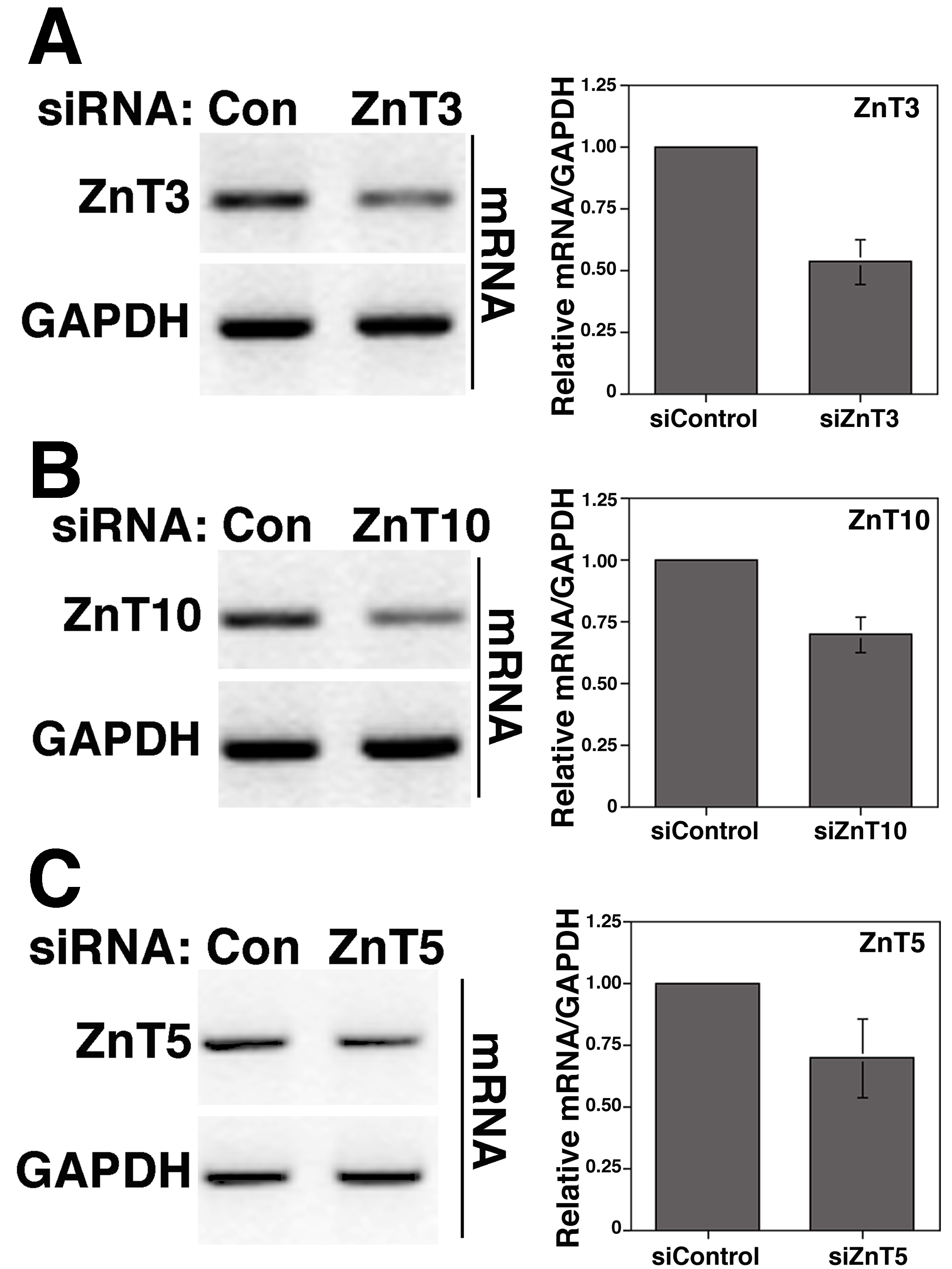

Supplement: Figure S2 — Expression of ZnT3, ZnT5 and ZnT10 decrease after siRNA treatment. Cells were incubated with siRNAs to downregulate ZnT3 (A), ZnT10 (B) or ZnT5 (C). Samples were separated in agarore gels and images inverted using Photoshop software. Relative mRNA levels were calculated with respect to GAPDH expression. **: p<0.01. (TIF) [file pone.0033211.s002.tif]

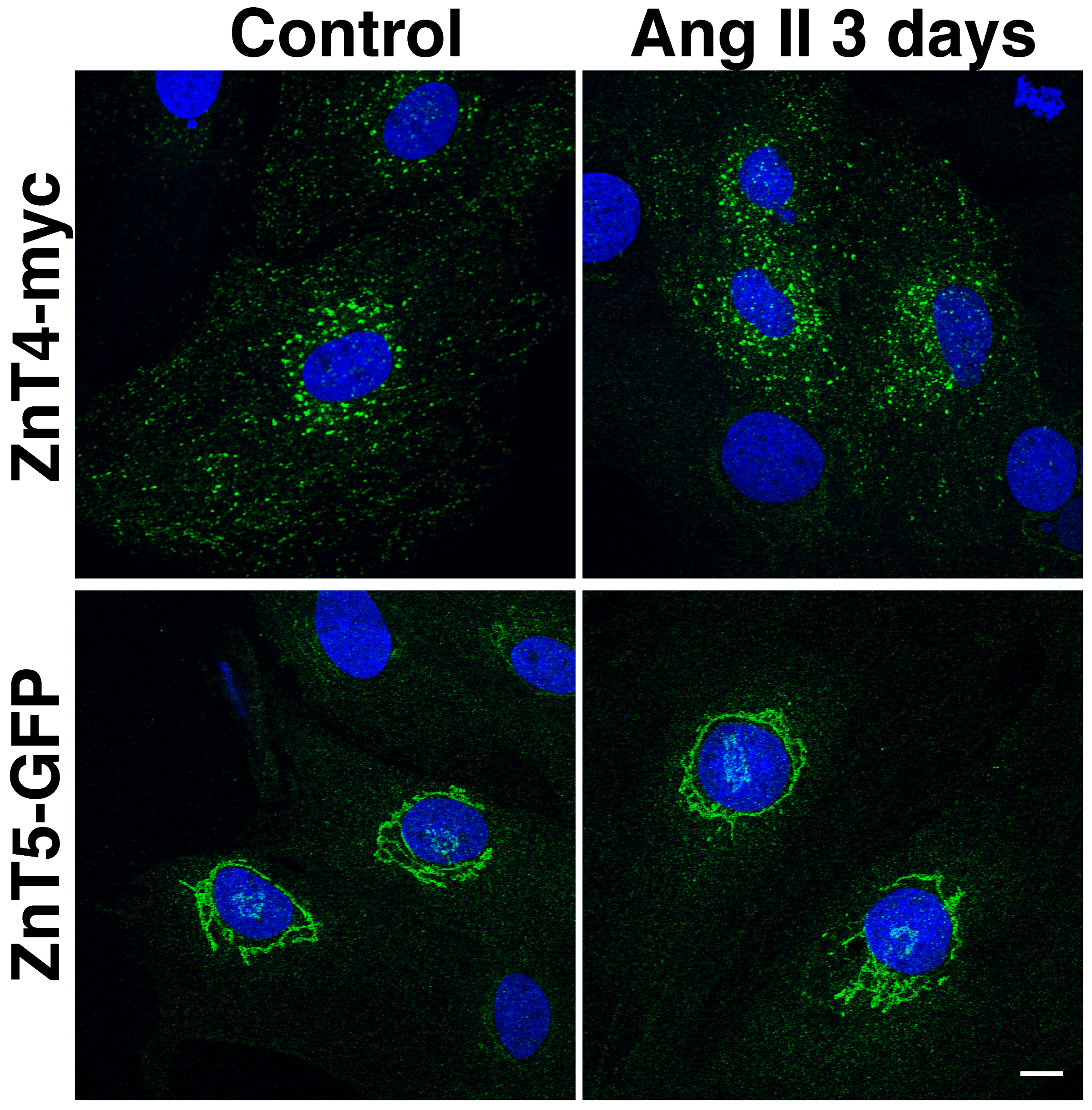

Supplement: Figure S3 — Subcellular localization of ZnT4 and ZnT5 is not affected by Ang II in VSMCs. Cells transfected with ZnT4-myc or ZnT5-GFP plasmids were incubated with or without Ang II for three days. Cells were fixed and incubated with anti-myc or anti-GFP antibodies. Images were acquired using a confocal microscope. Bar = 10 µm. (TIF) [file pone.0033211.s003.tif]

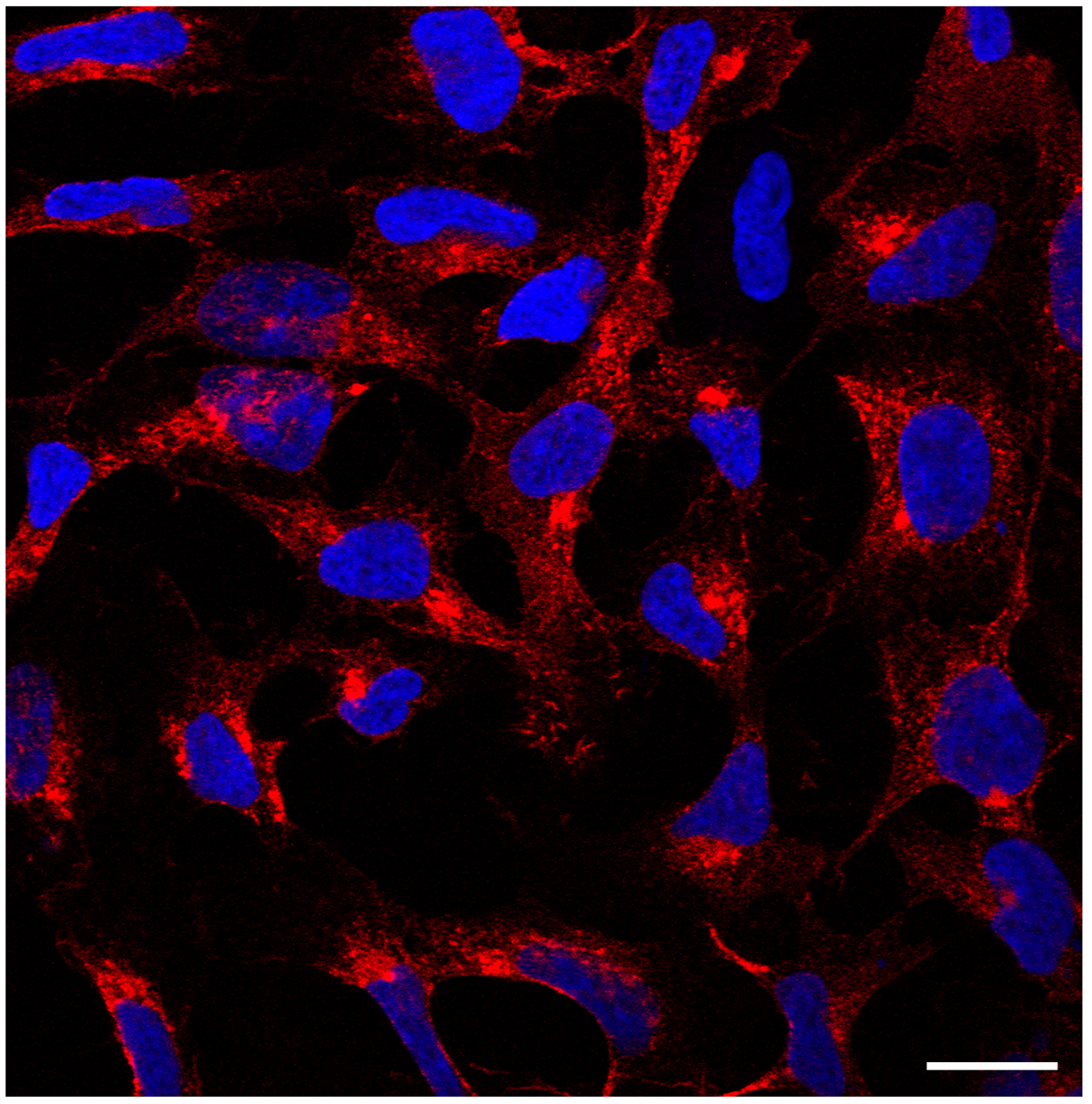

Supplement: Figure S4 — Expression of ZnT10-myc in transfected HEK293 cells. Permanently transfected HEK293 cells (colony N°18) were fixed with 4% PFA and incubated with polyclonal anti-myc antibodies. Alexa Fluor® 568 was used as a secondary antibody. Samples were imaged using a confocal microscope. Bar = 10 µm. (TIF) [file pone.0033211.s004.tif]

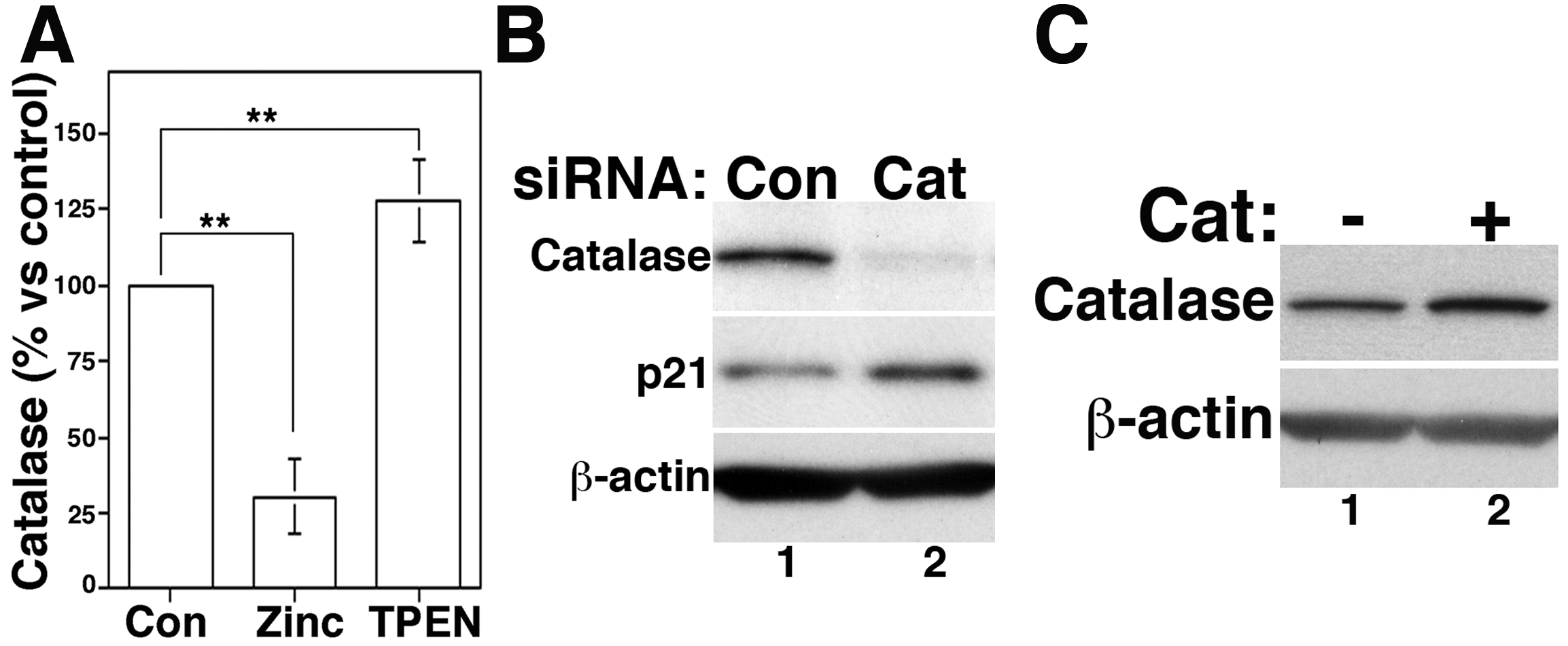

Supplement: Figure S5 — Catalase expression is downregulated by zinc and siCatalase. VSMCs were incubated with 50 µM zinc or 100 nM TPEN for five days (A), siCatalase (B) or transfected with plasmids containing catalase (C). Data on A represent quantification of catalase expression shown in Fig. 6A, expressed as percent versus control. **: p<0.01. (TIF) [file pone.0033211.s005.tif]

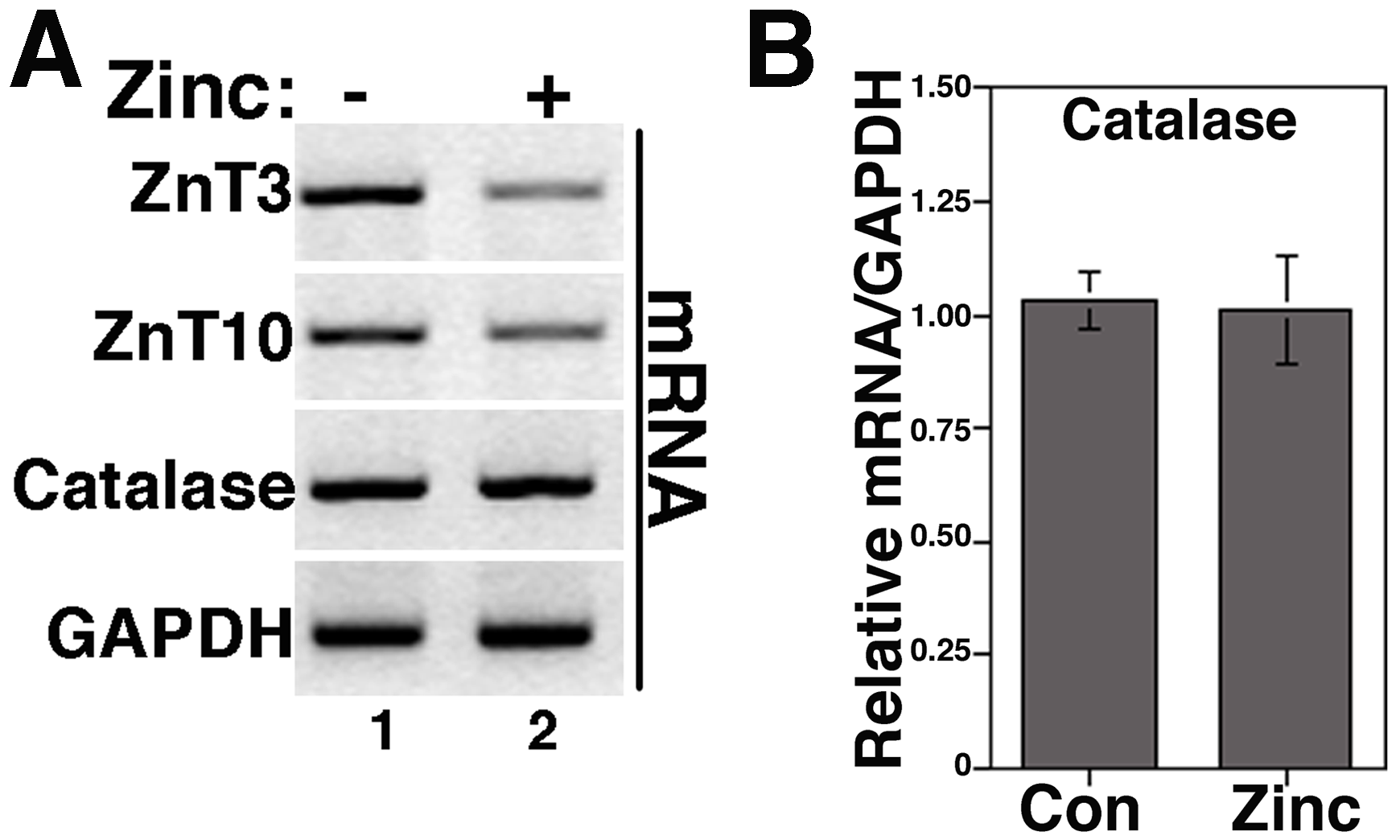

Supplement: Figure S6 — Zinc downregulates catalase by a post-transcriptional mechanism. A) Catalase mRNA levels were determined by RT-PCR after treatment with or without 50 µM zinc for three days. B) Catalase relative mRNA was calculated respect to GAPDH expression. (TIF) [file pone.0033211.s006.tif]

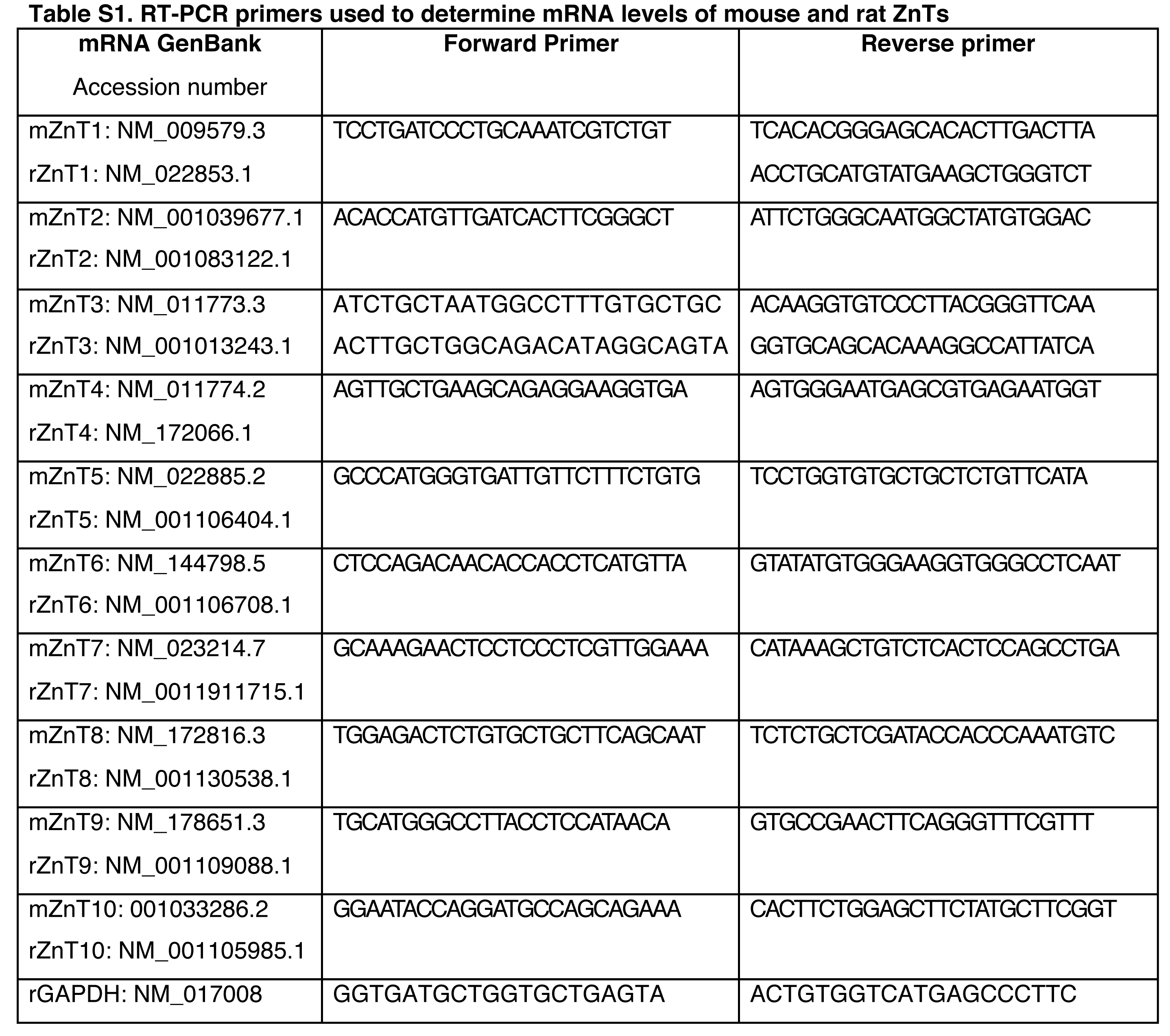

Supplement: Table S1 — Primers used to determine mRNA levels of mouse (m) and rat (r) ZnTs by RT-PCR. Primers were design to recognize mouse and rat ZnTs, except for ZnT1 and ZnT3. (TIF) [file pone.0033211.s007.tif]

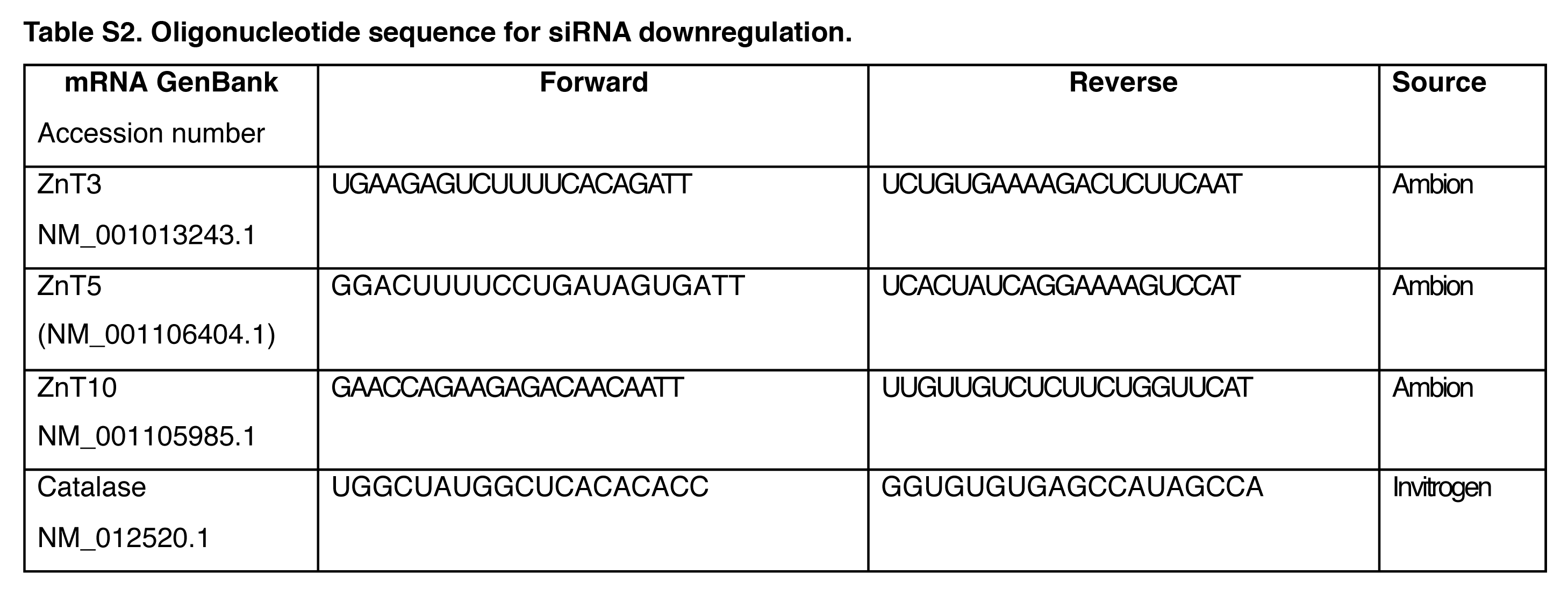

Supplement: Table S2 — Oligonucleotide sequence for siRNA downregulation. (TIF) [file pone.0033211.s008.tif]
